# Coping with Multiple Virulence Factors: Which Is Most Important? 

**DOI:** 10.1371/journal.ppat.0010040

**Published:** 2005-12-30

**Authors:** Erin E McClelland, Paul Bernhardt, Arturo Casadevall

Despite being a time when genomics and proteomics are becoming popular modes of scientific inquiry, most microbe-centric researchers continue to use reductionism to study virulence by identifying the microbial characteristics associated with virulence and characterizing each independently. Reductionism is a critical and remarkably powerful tool for determining the contribution of a single virulence factor to the overall virulence phenotype. However, the approach has the following limitation: most pathogenic microbes possess numerous attributes that contribute to virulence, and this virulence is a microbial trait that is expressed only in a susceptible host.

Unfortunately, it is often very difficult, if not impossible with current technology, to study combinations of virulence factors simultaneously, especially at the molecular level. Hence, new tools are needed to study the larger picture of virulence in a pathogenic organism and to better understand how the host and microbe interact. And while computer modeling, genomics, and proteomics contribute much to the larger picture of virulence, a more accessible method is available via multivariate statistics.

Consider two microbes: *Cryptococcus neoformans* and *Bacillus anthracis*. For *C*. *neoformans,* at least a half-dozen cellular attributes have been associated with virulence, including the polysaccharide capsule, melanin production, phospholipase secretion, mating factor, laccase, and urease [1]. Similarly, for *B*. *anthracis* the poly-D-glutamic acid capsule, lethal toxin, edema factor, and anthrolysin are each associated with the virulence phenotype [2,3]. Since immune responses to virulence factors often negate the virulence phenotype, vaccines often target virulence factors, as is the case for both *C*. *neoformans* and *B*. *anthracis*. In addition, the highly successful vaccines to *Streptococcus pneumoniae* and *Haemophilus influenzae* type B elicit antibodies to their polysaccharide capsules. In the same way, toxoid vaccines protect against tetanus and diphtheria by eliciting neutralizing antibody responses. Consequently, an investigator may want to prioritize the relative importance of virulence factors that contribute to the overall virulence phenotype, especially when designing new vaccines or antimicrobial drugs. However, to our knowledge, no methods have been developed to accomplish this.

Fortunately, the goal of prioritizing virulence factors for an individual microbe is similar to problems that have been encountered and solved in other disciplines where investigators have had to confront phenomena caused by multiple components. Sophisticated statistical methods have been developed to approach these problems and are applicable to the problem of microbial virulence. A commonly used statistical tool is multivariate linear regression analysis (MLRA), which has found a myriad of uses in the social sciences, biology, and medicine. For example, MLRA has been used to study aspects of school readiness such as the prediction of learning-related skills in children [4], factors that contribute to smoking in youths [5], the degree and location of white matter changes in patients with Alzheimer disease [6], and whether hand lead contamination is associated with blood lead contamination [7]. MLRA simultaneously assesses the relationships of many independent variables to one dependent variable, and can easily be used to examine the relative contribution of microbial virulence factors to virulence. In fact, MLRA can be used to rank virulence factors in disease importance.

There are three different kinds of MLRA: standard multiple regression, sequential (hierarchical) regression, and statistical regression [8]. In standard multiple regression, all independent variables are entered together so that the relative contribution of each independent variable to the dependent variable is assessed at the same time. Thus, standard regression illustrates how much of the dependent variable is explained by each of the independent variables at once. In hierarchical regression, the independent variables are entered in different steps in a specific order, with the order of entry resulting from theoretical or logical importance. Thus, hierarchical regression allows the investigator to examine the unique contribution of each independent predictor variable to the variance in the dependent variable, while taking into account the contribution of previously identified independent variables. In statistical regression, the independent variables are entered or removed in different steps, in an order that is specified by statistical criteria. Thus, statistical regression is useful for selecting which independent variables best predict the dependent variable when there is no theoretical rationale for a priori prioritization [8]. Depending on how much information is available on certain microbial virulence factors, and the focus of the research question, investigators can use any or all of the different kinds of MLRA.

One limitation of MLRA is the need for an adequate sample size. A general rule of thumb is that ten data points (or microbial strains) are needed for every independent variable. So if the microbe of choice has three virulence factors, a sample size of at least 30 strains demonstrating some variation in virulence and virulence factor expression are needed to ascertain the relative contribution of each virulence factor to the overall virulence phenotype. However, Maxwell estimates that this rule of thumb is overly optimistic [9]. A more conservative estimate is 20–30 data points for every independent variable, based on the correlation between the independent variables (EEM, PB, and AC, unpublished data). More data points are needed if the independent variables are more correlated to each other. In addition, the sample size required is dependent on the question asked. A smaller sample size is needed if the goal is to establish which virulence factor contributes most to virulence (a large effect, such as which virulence factor is the most important candidate for drug and vaccine development research). However, a much larger sample size is needed if the investigator is looking for smaller effects, such as how much each microbial virulence factor contributes to virulence.

Another consideration is the measure of virulence that will be used as the dependent variable. Such measures could include survival, microbial numbers in tissue, as well as different measures of host damage and the immune response [10]. Alternatively, strains could be tested for virulence in alternate hosts such as *C*. *elegans* and amoeba, since many microbial virulence factors can induce damage in multiple hosts and may be evolutionarily conserved [11].

The use of this method requires knowledge of statistics or collaborations with statisticians. As the average microbiologist may not be statistically trained, and statisticians that are familiar with experimental biological methods are often few and far between, it is our opinion that this could be addressed by requiring a statistics course for any graduate program, including a microbiology PhD program or medical school. Not only will every scientist benefit from statistical training with regard to experimental design and planning for statistical power, but because statistics is not often stressed in certain scientific disciplines, many journals are now requiring that statistical analysis be part of the methods section of submitted manuscripts. Thus, statistical training is an increasingly important requirement for every scientist.

We have proposed that for microbes with multiple virulence factors, MLRA can be used to explore their contribution to virulence, with the caveat that investigators need to use an appropriate sample size for confident predictions. Information gleaned from estimates of the relative contribution of virulence factors to the larger picture of virulence could be exploited for selecting targets for drug design and vaccines. 

## 

**Figure:**
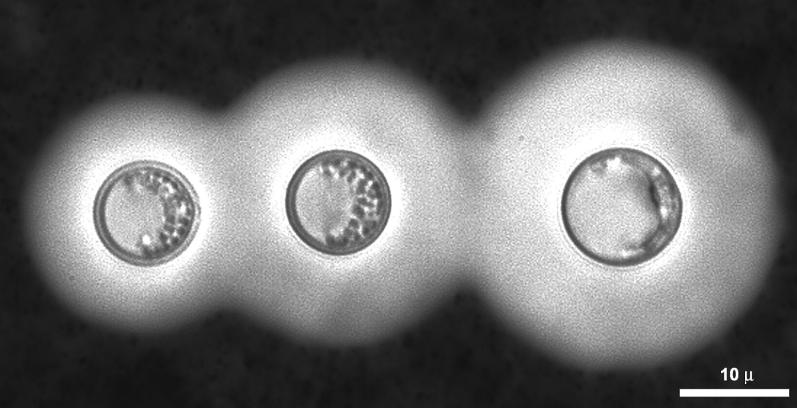
**India ink of *C. neoformans***

## Abbreviation

MLRA: multivariate linear regression analysis
